# Cancer risk in children and young adults born preterm: A systematic review and meta-analysis

**DOI:** 10.1371/journal.pone.0210366

**Published:** 2019-01-04

**Authors:** Katryn Paquette, Hallie Coltin, Ariane Boivin, Devendra Amre, Anne-Monique Nuyt, Thuy Mai Luu

**Affiliations:** 1 Department of Pediatrics, Sainte-Justine University Hospital and Research Center, University of Montreal, Montreal, Quebec, Canada; 2 Department of Pediatrics, Children’s Hospital of Eastern Ontario, University of Ottawa, Ottawa, Ontario, Canada; 3 CEGEP Garneau, Quebec City, Quebec, Canada; Universita degli Studi di Milano, ITALY

## Abstract

**Introduction:**

Risk of developing a malignancy when born premature is unknown. We hypothesised that risk of certain cancers might be increased in youth born preterm versus term. We therefore performed a systematic review and meta-analysis to evaluate the incidence of malignancy in the context of preterm birth, according to various cancer types.

**Methods:**

The study was designed per MOOSE and PRISMA guidelines. Articles were identified through November 2015. Observational studies exploring the association between childhood malignancy and birth characteristics were included. Of the 1658 records identified, 109 full text articles were evaluated for eligibility. Random effects meta-analyses were conducted on 10/26 studies retained; 95% confidence intervals were computed and adjusted following sensitivity analysis. Publication bias was evaluated using funnel plots, Begg’s and Egger’s tests.

**Results:**

No differences in risk of primary central nervous system tumor [OR 1.05; 95% CI 0.93–1.17, 5 studies, 580 cases] and neuroblastoma [OR 1.09; 95% CI 0.90–1.32, 5 studies, 211 cases] were observed in individuals born <37 versus ≥37 weeks’ gestation. Preterm birth was consistently associated with hepatoblastoma [ORs 3.12 (95% CI 2.32–4.20), 1.52 (95% CI 1.1–2.1), 1.82 (95% CI 1.01–3.26), and 2.65 (95% CI 1.98–3.55)], but not leukemia, astrocytoma, ependymoma, medulloblastoma, lymphoma, nephroblastoma, rhabdomyosarcoma, retinoblastoma or thyroid cancer.

**Conclusions:**

Children born premature may be at increased risk for hepatoblastoma but there is no strong evidence of an increased risk of primary central nervous system tumours or neuroblastoma. There is insufficient evidence to conclude whether prematurity modulates the risk of other childhood cancers.

## Introduction

Perinatal characteristics have been described as independent risk factors for cancer development. An estimated 10% of neonates are born preterm; more than 95% of these babies live[1]. In preterm newborns, significant insults result from the exposure of immature antioxidant defence systems to pro-oxidants, such as oxygen, UV light, parenteral nutrition, and plastic derivatives[2]. Oxidative stress has been established in the causal pathway of two common short-term sequelae of prematurity, bronchopulmonary dysplasia and retinopathy of prematurity, as well as death[3–5].

Experimental data suggest that enduring impacts of early oxidative stress exposure include premature arrest of cellular proliferation, rapid tissue differentiation, oxidative DNA damage, and impaired DNA repair[6–8]. As a result, preterm birth might increase the risk of cancer development. Researchers have attempted to evaluate the impact of perinatal factors on cancer development, but have been unable to isolate for the effects of gestational age (GA) with sufficient power to risk stratify for prematurity. As a result, the effect of low birth weight and intrauterine growth restriction on the risk of cancer are the focus of the majority of published review papers. Two recent meta-analyses suggested that prematurity augments the risk of nephroblastoma and testicular cancers, but were hampered by publication bias and divergent methodologies within the observational studies used in their summary effects[9, 10]. Moreover, these cancers comprise <6% of all pediatric oncologic cases[11]. The risk afforded by prematurity for developing childhood cancer thus remains unclear.

We therefore conducted a systematic review and meta-analysis of observational studies to determine whether preterm birth was associated with increased incidence of childhood malignancy, according to cancer type. We hypothesised that the risk of certain cancers may be increased in children and young adults born preterm versus term.

## Materials and methods

We designed the study per MOOSE and PRISMA guidelines. Articles were identified from EMBASE, Medline (including In-Process & Other Non-Indexed Citations), and all EBM Reviews (Cochrane DSR, ACP Journal Club, DARE, CCTR, CMR, HTA, and NHSEEDE) by the principal investigator (KP) from the inception date of each resource to November 1, 2015. Expandable terms were used as MESH headings and keywords: cancer, malignan*, leukemia or leukaemia, lymphoma, tumor or tumour, *blastoma, *sarcoma, *carcinoma*. Any of these terms were combined with ((prem* or preterm) and birth)) and (infant or child* or juvenile). The search was limited to children (0–21 years) and writings in English or French. Reference lists were searched for additional titles (S1 Table). Authors were not contacted for unpublished work they may be aware of. Search was designed by KP in association with institution’s librarians.

Regarding article selection, original observational studies conducted on humans were considered if they reported the rate of childhood cancers in the context of GA at birth. Articles were excluded if the specific GA could not be ascertained, the definition of prematurity was not <37weeks GA, the method for cancer diagnosis was not stated or was not an accepted standard at the time of diagnosis, if there was a lack of term-born controls, and if all the reported cases were included in another study from our data set. Articles pertaining to testicular tumours were excluded, as a recent meta-analysis examined their association with GA[10].

All titles and abstracts were independently reviewed for eligibility by two reviewers (AB, KP) who then assessed full-text articles per selection criteria. Disagreements were resolved by consensus. Data extraction and quality assessment were performed by three independent individuals (AB, HC, KP). The quality of each article was objectively appraised using the Newcastle-Ottawa Scale (NOS) for Assessing the Quality of Nonrandomized Studies in Meta-Analysis.

Data was extracted by three independent authors (KP, HC, AB) for study methodology, publication date, cancer type, country(ies) included, year(s) of diagnosis, age at time of diagnosis, method of diagnosis, study population, matching criteria, confounders, risk estimates by GA, and the use of adjustment models. Two authors were contacted for additional information. Although these authors responded to our queries, the details that we requested were not available. Extracted data were entered into Microsoft Excel Version 1704 (Microsoft, Redmond, WA, USA). The data underwent a final review by two authors (KP, DA) prior to analysis to assess and correct for discrepancies.

Meta-analysis was performed in Stata/IC 12.1 (Stata Corp, College Station, TX, USA). Analyses were performed using odds ratios (ORs) per unadjusted risk estimates reported in the primary publications for birth at < vs ≥37 weeks GA. In the absence of unadjusted risk estimates for these dichotomized GA categories, they were calculated from frequency tables provided in the manuscript or by personal correspondence with study investigators. Unadjusted values were used to mitigate the heterogeneity induced by combining risks adjusted for different characteristics and to allow for a larger sample size (multiple articles retained did not have adjusted risk estimates). However, repeating analyses using adjusted risk estimates when available altered neither the magnitude nor the direction of the summary effects.

To account for inter-study variability and increase meta-analysis power, outcomes with five or more studies were subjected to random effects meta-analyses. Random effects meta-analyses were used due to the inherent heterogeneity present between observational studies. Outcomes with four or fewer studies were assessed from a qualitative perspective only. Due to its rare occurrence, pooled analyses stratifying cancer risks by the degree of prematurity were not performed. Heterogeneity was evaluated using the I^2^ statistic. Finally, publication bias was evaluated by inspection of funnel plots, Egger’s and Beggs’ tests.

## Results

**Fig 1:**
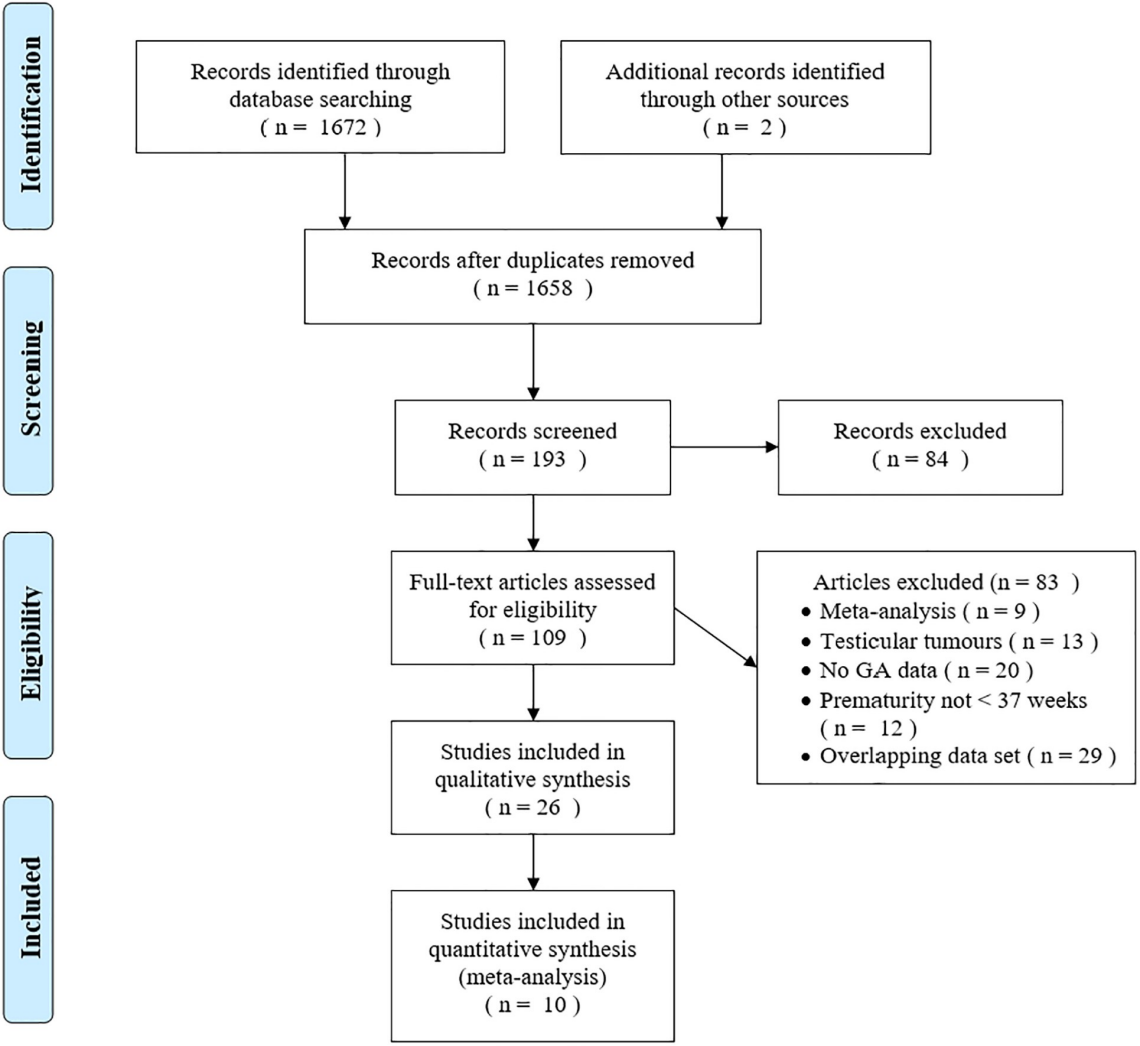
PRISMA flow diagram describing study selection process.

The database search identified 1,658 records; 193 remained after screening their titles. Of these, 84 studies were excluded after reading their abstracts. We evaluated 109 full text articles for eligibility, 26 were retained for the qualitative synthesis, of which 10 were eligible for meta-analysis (Fig 1). We excluded 29 articles with overlapping data sets. When multiple studies had overlapping populations, we included the study, or groups of studies, with the largest sample size. Of the 26 studies retained, 13 were of good quality (NOS 4–6 stars)[12–24] and 13 were excellent (7–9 stars)[25–37]. See Table 1 for study details. See Table 2 for summary of non-meta-analyzed cancer-specific data.

**Table 1:** Studies retained for the meta-analysis and systematic review.

Studies Retained for Meta-Analysis							
Author, year	NOS^a^	Study Design	Data Source	Country ± State	Years of Diagnosis	Cancer Type	Age at Diagnosis
Emerson, 1991	7	Case control	Registry	USA: WA^b^	1974–1986	CNS^c^ tumors	≤10 years
Buck, 2001	5	Case control	Registry Medical Records Phone Interview	USA: NY^d^	1976–1987	Neuroblastoma	≤5 years
Fear, 2001	4	Case control	Medical Records	UK	1956–1992	CNS tumors	≤14 years
Hamrick, 2001	6	Case control	Registry Phone Interview	USA, Canada	1992–1994	Neuroblastoma	≤18 years
Bluhm, 2008	6	Nested Case control	Registry	Sweden	1973–1995	Neuroblastoma	≤21 years
Munzer, 2008	5	Case control	Phone Interview Interview at diagnosis	France	2003–2004	Neuroblastoma	≤14 years
Spector, 2009	7	Case control	Registry	USA: CA^e^, MN^f^, NY, TX^g^, WA	1980–2004*	All common malignancies	28 days– 14 years^§^
Schmidt, 2010	7	Case control	Registry	Denmark Finland Norway Sweden	1985–2006	CNS tumors	≤14 years
Sprehe, 2010	6	Case control	Registry	USA: TX	1995–2003	Cancers, leukemias, CNS tumors	≤4 years
Oksuzyan 2013	7	Case control	Registry	USA: CA	1988–2008	CNS tumors	≤15 years
**Studies Retained for Qualitative Analysis Only**							
Hjalgrim, 2004	8	Case control	Registry	Denmark, Iceland, Norway, Sweden	1984–1999	ALL^h^	≤14 years
Jepsen, 2004	6	Case control	Registry	Denmark	1973–1993	Wilms Tumor	≤14 years
Podvin, 2006	6	Case control	Registry	USA: WA	1981–2008	Leukemia–all combined	≤19 years
Johnson, 2011	7	Case control	Registry	USA: CA, MN, NY, TX, WA	1980–2004	All carcinomas	28days– 14 years
Schüz, 2011	7	Case control	Registry	Denmark Finland Norway Sweden	1985–2006	Wilms tumor	≤14 years
Crump, 2012	8	Cohort	Registry	Sweden	1973–2009	Hodgkin Lymphoma	1–37 years*
Crump, 2012	8	Cohort	Registry	Sweden	1973–2009	Non-Hodgkin Lymphoma	0–37 years*
de Fine Licht, 2012	7	Case control	Registry	Denmark Finland Norway Sweden	1985–2006	Hepatoblastoma	≤14 years
Heck, 2012	6	Case control	Registry	USA: CA	1988–2007	Retinoblastoma	≤5 years
Oksuzyan, 2012	7	Case control	Registry	USA: CA	1988–2008	Leukemias	≤15 years
Puumala, 2012	6	Case control	Registry Phone Interview	USA	2000–2008	Hepatoblastoma	≤5 years
Heck, 2013	6	Case control	Registry	USA: CA	1988–2007	Hepatoblastoma	≤5 years
Shrestha, 2013	6	Case control	Registry	USA: CA	1988–2008	Rhabdomyosarcoma	≤5 years
Crump, 2014	8	Cohort	Registry	Sweden	1973–2009	Wilms tumor	?
Marcotte, 2014	6	Case control	Registry	USA: CA	1988–2007	Lymphomas	≤5 years
Crump, 2015	8	Cohort	Registry	Sweden	1973–2010	ALL	0–≥15 years^∞^

^a^Newcastle Ottawa Score for quality,

^b^Washington State,

^c^Central Nervous System,

^d^New York State,

^e^California,

^f^Minnesota,

^g^Texas,

^h^Acute Lymphoblastic Leukemia

*CA 1988–1997, MN 1988–2004; NY 1985–2001; TX 1990–1998; WA 1980–2004

^§^CA 28days– 4 years

^∞^5% of cases adults, 11% of cases ≥16years old

**Table 2:** Cancer-specific data not amenable to meta-analysis.

Author, year	Cases Born < 37 Weeks	Cases Born ≥ 37 Weeks	Controls Born < 37 Weeks	Controls Born ≥ 37 Weeks	Odds Ratio (95% Confidence Interval)
Acute Lymphocytic Leukemia					
Hjalgrim, 2004	186	1,631	846	7,981	1.08 (0.91–1.27)
Spector, 2009*	371	4,199	4,404	50,858	1.02 (0.91–1.14)
Oksuzyan, 2012	466	4,005	441	3,987	1.05 (0.92–1.21)
Crump, 2015	111	1,849	205,387	3,362,946	0.98 (0.81–1.19)
Acute Myeloid Leukemia					
Hjalgrim, 2004	26	254	124	1,248	1.03 (0.66–1.61)
Spector, 2009*	61	758	4,404	50,858	0.93 (0.71–1.21)
Oksuzyan, 2012	120	681	87	717	1.45 (1.08–1.95)
All Leukemias Combined					
Podvin, 2006	42	535	437	5,279	0.96 (0.69–1.34)
Sprehe, 2010	253	2090	1,301	10,982	0.92 (0.73–1.16)
Oksuzyan, 2012	642	5145	573	5215	1.14 (1.00–1.28)
Astrocytoma					
Emerson, 1991	5	NR^§^	35	NR	0.70 (0.20–1.70)
Spector, 2009*	108	1495	4,404	50,858	0.78 (0.58–1.08)
Oksuzyan, 2013	111	1176	132	1,157	0.83 (0.63–1.08)
Ependymoma					
Emerson, 1991	3	NR	3	NR	5.5 (1.0–29.5)
Spector, 2009*	24	355	4,404	50,858	0.90 (0.60–1.90)
Oksuzyan, 2013	23	237	30	228	0.73 (0.41–1.29)
Medulloblastoma					
Emerson, 1991	33	NR	13	NR	1.2 (0.30–4.7)
Oksuzyan, 2013	44	324	39	330	1.16 (0.73–1.84)
Nephroblastoma					
Jepsen, 2004	7	119	NR	NR	1.05 (0.43–2.56)
Spector, 2009*	120	1023	4,404	50,858	1.35 (1.19–1.64)
Schüz, 2011	39	627	148	3017	1.27 (0.88–1.82)
Crump, 2014	31	376	206,813	1,503,188	1.22 (0.85–1.76)
Non-Hodgkin Lymphoma					
Spector, 2009*	47	522	4,404	50,858	1.04 (0.77–1.40)
Crump, 2012	54	882	206,790	3,363,748	1.00 (0.76–1.31)
Marcotte, 2014	14	156	20,118	176,842	0.78 (0.45–1.34)
Hodgkin Lymphoma					
Spector, 2009*	34	431	4,404	50,858	0.91 (0.64–1.29)
Crump, 2012	54	871	206,790	3,363,841	0.98 (0.75–1.30)
Marcotte, 2014	1	55	20,118	176,842	-
All Lymphomas Combined					
Spector, 2009*	104	1,153	4,404	50,858	1.04 (0.85–1.28)
Marcotte, 2014	45	410	20,118	176,842	0.95 (0.70–1.29)
Hepatoblastoma					
Spector, 2009*	56	207	4,404	50,858	3.12 (2.32–4.20)
Puumala, 2012	87	294	119	265	1.52 (1.1–2.1)
de Fine Licht, 2012	17	87	49	650	1.82 (1.01–3.26)
Heck, 2013	59	189	21,749	184,715	2.65 (1.98–3.55)
Rhabdomyosarcoma					
Spector, 2009*	42	522	4,404	50,858	0.93 (0.8–1.27)
Shrestha, 2013	36	309	20,099	176,647	1.04 (0.73–1.46)
Retinoblastoma					
Spector, 2009*	64	603	4,404	50,858	1.23 (0.95–1.58)
Heck, 2012	61	548	21,323	187,728	1.02 (0.77–1.34)
Thyroid Cancer					
Johnson, 2011	18	123	NR	NR	1.87 (1.07–3.27)

*Spector et al recruited 4,404 preterm-born and 50,858 term-born children who were cancer free between 28 days and 15 years of age; these children represented the preterm-born controls for all cancers studied.

^§^ Not reported

### Leukemia

Overall, six studies reported on leukemias, including one from Oksuzyan et al [31], which did not exclude individuals with trisomy 21, a major risk factor for leukemias in general. Indeed, Oksuzyan et al were the only authors to find difference in leukemias in preterm versus full-term born individuals (see below).

Four manuscripts combining 1,134 patients born preterm were retained to evaluate the relationship between gestational age and ALL [30, 31, 35, 36]. The risk of ALL was similar between studies; these studies did not suggest an association between ALL and preterm birth (Table 2). Data were not meta-analyzed due to population overlap.

Three studies compared AML in individuals born at <37 versus ≥37 weeks, totalling 245 cases [30, 31, 35] (Table 2). Two studies highlighted no difference in the risk of AML when born preterm, while the study by Oksuzyan et al suggested an increased risk (OR 1.45; 95% CI 1.08–1.95)[31]. No summary effects were performed due to the limited sample of studies.

Three studies assessed risks of any leukemia following preterm birth [20, 21, 31] (Table 2). These studies yielded 937 exposed cases with an association between preterm birth and leukemia only identified in Oksuzyan et al (OR 1.14; 95% CI 1.00–1.28)[31].

### Primary CNS tumors

Six studies evaluated the association between premature birth and primary CNS tumors [14, 21, 29, 32, 33, 35]. These were case control studies with minimal population overlap; most included children from birth to adolescence.

**Fig 2:**
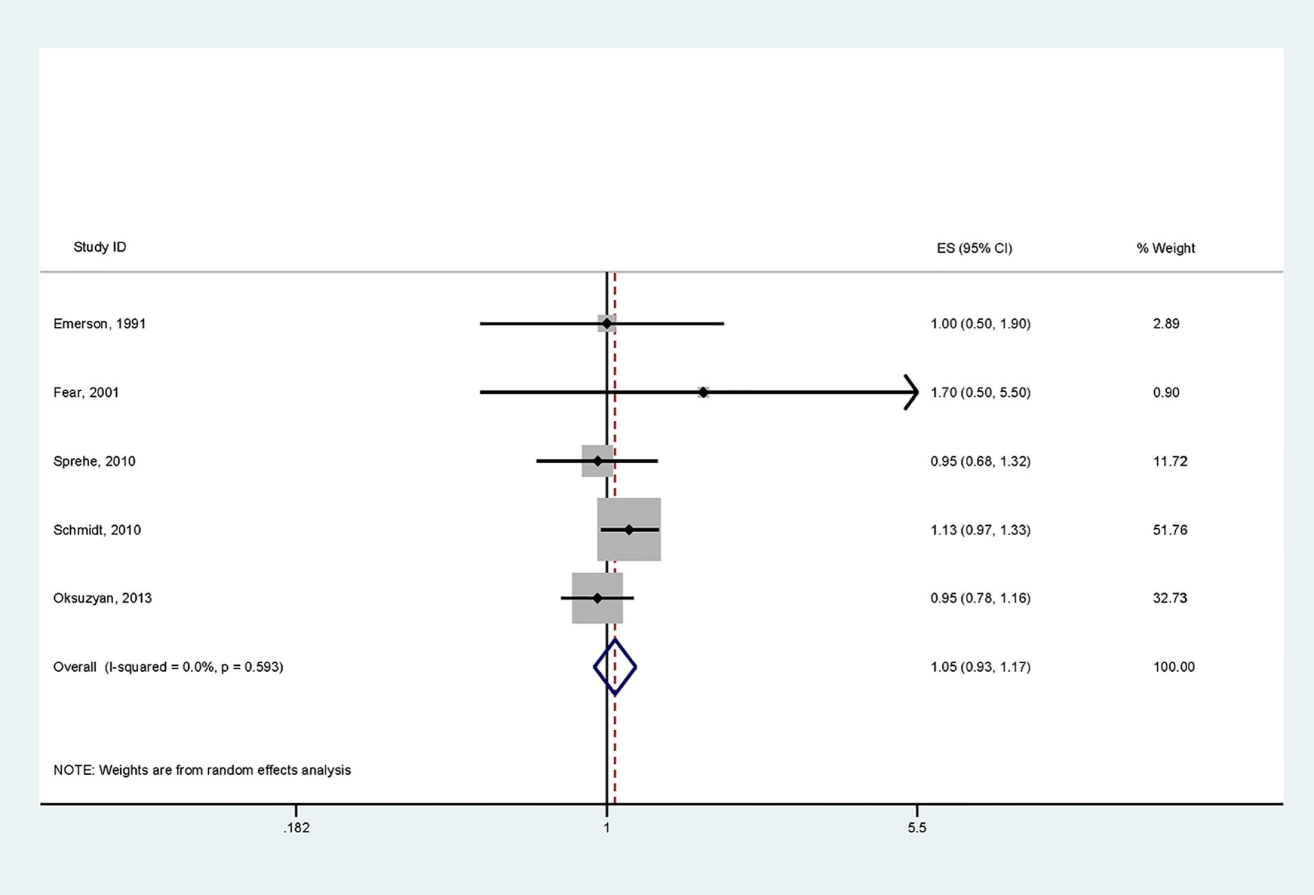
Forest plot of GA < vs ≥37weeks and the risk of developing a primary central nervous system tumor.

Five manuscripts reported ORs for all primary CNS tumors combined [14, 21, 29, 32, 33]. All five studies were case control by design without overlap in study region. There was, however clinical heterogeneity in the underlying study designs: Emerson et al, Shmidt et al and Oksuzyan et al had no exclusion criteria, while Fear et al excluded children born with physical or chromosomal abnormalities and Sprehe et al excluded children born of multiple gestation pregnancies (i.e. not singletons). Furthermore, the age at diagnosis of children included in the studies varied between ≤4 years and ≤15 years of age. Combining their data yielded 580 cases and a summary OR of 1.05 (95% CI 0.93–1.17) in those born at <37 versus ≥37 weeks (Fig 2). Despite the clinical differences in study inclusion criteria, no heterogeneity (I^2^ 0%) or publication bias (S1 Fig) were noted in the summary effects.

Risk assessments for individual primary CNS tumors were reported by three studies [29, 32, 35]. There was no significant difference in risk for astrocytoma or medulloblastoma (Table 2). Fifty cases of ependymoma in individuals born preterm were reported yielding ORs of 5.5 (95% CI 1.0–29.5)[29], 0.90 (95% CI 0.60–1.90)[35], 0.73 (0.41–1.29)[32]. The study by Emerson et al, the only study to suggest that birth at ˂37weeks was associated with an increased risk of ependymoma when compared to birth at ≥ 37 weeks, was the oldest study in this set, including children diagnosed between 1974 and 1986. Spector et al and Oksuzyan et al recruited from the 1980s through to the 2000s. Furthermore, Emerson et al included children diagnosed at ≤ 10 years of age, whereas latter two teams recruited adolescents up to 14 and 16 years of age respectively. All three studies were of American youth.

### Neuroblastoma

**Fig 3:**
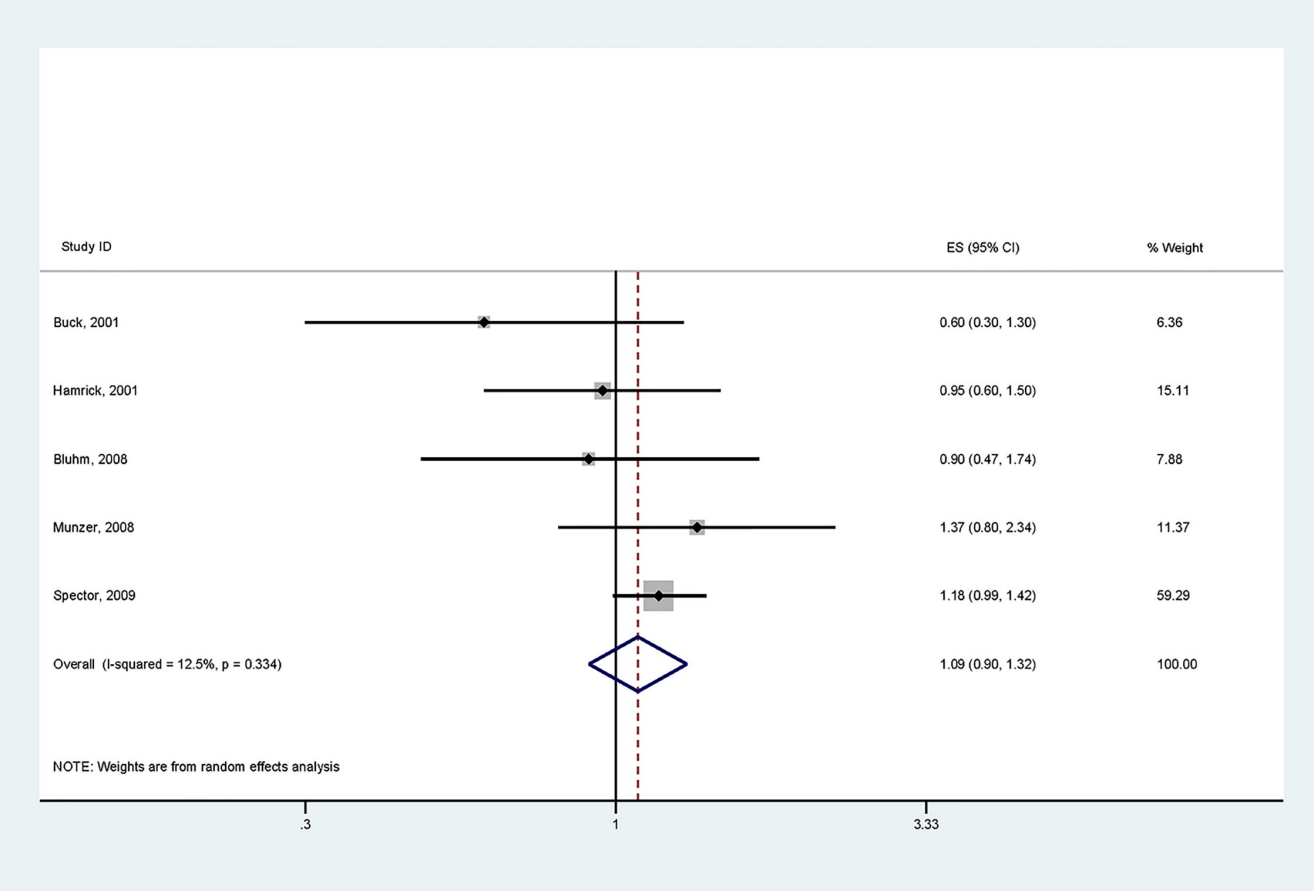
Forest plot of GA < vs ≥37 weeks and the risk of developing neuroblastoma.

Five articles were retained pertaining to neuroblastoma [12, 13, 15, 19, 35]. Of the 211 cases among children born preterm, 87% were North American. The studies’ background methodologies were homogeneous: case control designs, no exclusion criteria except for Spector et al (trisomy 21), and most recruiting patients into adolescence. Risk of neuroblastoma was not increased in those born <37 versus ≥37 weeks GA (OR 1.09; 95% CI 0.9–1.32). For the summary effect, heterogeneity was minimal with I^2^ 12.5% (Fig 3). No publication bias was noted (S2 Fig).

### Nephroblastoma

Four studies remained for the analysis of nephroblastoma risk [18, 25, 34, 35] (Table 2). Two were case control by design, all were registry based, and 61% of the 197 cases were from the United States. Due to significant case duplication and the small sample size, summary effects were not calculated. Only Spector et al found significant difference in nephroblastoma in preterm versus full-term born individuals (OR 1.35; 95% CI 1.19–1.64)[35].

### Lymphoma

Four heterogeneous studies evaluating the development of lymphoma were retained[22, 26, 27, 35]. Indeed, both studies by Crump et al were cohorts that included persons ≤36 years old. Spector et al conducted a case control study recruiting children 28 days to 14 years old. Finally, Marcotte et al performed a case control study recruiting preschoolers ≤4 years old. Due to the heterogeneity in study methodology and limited sample size, no summary effects were calculated. Non-Hodgkin lymphomas, Hodgkin lymphomas, or any lymphomas were not significantly different in preterm versus full-term born individuals (Table 2).

### Hepatoblastoma

Four case control studies measured the association between prematurity and hepatoblastoma[17, 23, 28, 35]. Of the 219 cases among children born preterm, 92% were North American. Indeed, all studies but the one by de Fine Licht et al originated from the United States. All studies suggested that hepatoblastoma was more common following preterm versus full-term birth with ORs of 3.12 (95% CI 2.32–4.20)[35], 1.52 (95% CI 1.1–2.1)[23], 1.82 (95% CI 1.01–3.26)[28], and 2.65 (95% CI 1.98–3.55)[17].

### Other solid organ cancers

We found a small sample of studies on other solid organ cancers. Two studies reported findings for rhabdomyosarcoma[24, 35]. Spector et al included ≤14-year-olds in five states, while Shrestha et al recruited Californian children less than five years of age from 1988–2008. There was a 10-year overlap between studies. Seventy-eight cases were reported with ORs of 0.93 (95% CI 0.8–1.27)[35] and 1.04 (95% CI 0.73–1.46)[24] respectively.

Two registry-based case control studies with significant sample duplication evaluated the impact of prematurity on retinoblastoma[16, 35]. Heck et al reported 61 exposed Californian cases from 1988–2007, with an OR of 1.02 (95% CI 0.77–1.34) in the event of preterm birth. Spector et al found 64 cases in five states, including California (1988–1997), with an OR of 1.23 (95% CI 0.95–1.58).

Johnson et al described the prevalence of thyroid cancer in U.S. children. They reported 18 cases in individuals born preterm with OR of 1.87 (95% CI 1.07–3.27)[37].

## Discussion

Our results suggest that there is no significant difference in the risk of primary CNS tumors or neuroblastoma in those born preterm versus not preterm. Data was insufficient to conclude whether there is an impact on the development of leukemias, astrocytomas, ependymomas, medulloblastomas, nephroblastoma, lymphomas, rhabdomyosarcoma, retinoblastoma or thyroid cancer when born prematurely. Preterm birth may increase the risk of hepatoblastoma. Our results echo the findings of most published studies addressing this question.

A recent meta-analysis evaluating the impact of preterm birth on childhood leukemias also noted no increase in the risk of ALL or all leukemias combined, but found a modest increase in the risk of AML[38]. However, the studies included for summary effects were older and numerous samples were entirely duplicated. Conversely, Crump et al updated reports of AML cases in Sweden between 1973–2010[39]; 14 cases occurred in persons born preterm for an OR of 0.76 (95% CI 0.44–1.3), again shifting the balance of risk to one of neutrality.

Our results are also concordant with an updated case control study of adolescent CNS tumors in Scandinavia[40]. Among children born at <37 vs ≥37 weeks, the odds of developing a primary CNS tumor or astrocytoma were 1.04 (95% CI 0.48–2.3) and 0.62 (95% CI 0.19–1.99) respectively.

Unlike a 2010 meta-analysis[9], our data do not suggest that preterm birth increases the risk of nephroblastoma. We did not perform summary effects due to the limited sample size and high frequency of case duplication. Indeed, more than 75% of the weight for summary effects in the aforementioned manuscript arose from studies with methodological limitations: two defined preterm birth as <38 weeks, one did not define preterm birth. Since these three studies had the largest and most significant ORs [1.28–1.88 (95% CIs 0.94–1.27, 1.74–2.78)], the overall cancer risk was over-estimated.

Cancer development requires a complex mix of cellular dysregulation and aberrant immune surveillance within the host to enable significant proliferation of a malignant line. Mechanisms which have been implicated in childhood cancer include abnormal growth factor signaling pathways, ionizing radiation, oxidative stress, environmental toxin exposure, viral infection, and underlying genetic mutations or syndromes [41–43]. Although preterm neonates are confronted by many of these adverse elements in utero and then in the NICU, there is also evidence that ex-utero organogenesis results in reduced cellular endowment in many developing organs [44, 45]. A decreased pool for cellular proliferation may help counter pro-oncogenic factors, helping to mitigate the malignant potential otherwise introduced by premature birth.

It is biologically plausible that the liver remains a vulnerable organ with increased susceptibility to cancer when exposed to preterm birth. The newborn liver is a highly metabolic organ responsible for numerous endocrine and exocrine functions, while undergoing maturation[46, 47]. This organ is exposed to repeated stressors via low lying umbilical venous lines, parenteral nutrition and its peroxide load, the stress of polypharmacy, and the first pass effect. The first pass effect is significant, whereby the liver serves as a gateway, metabolizing ingested compounds prior to their entering the systemic circulation. Furthermore, potential hepatocarcinogens such as the plasticizer di-(2-ethylhexyl)phthalate[48], which has been associated with liver cancer in animal models, may also be present in the nursery causing exposure during infancy.

Our study’s findings carry strong internal and external validity due to rigorous methodology, the inclusion of high-quality studies, minimization of duplicated populations, and computation of risk effects with a precise and accurate definition of preterm birth. The studies we included spanned multiple decades and 75% recruited cases into the 2000s, reducing the population bias inherent to earlier meta-analysis. Older manuscripts included individuals born primarily before 1990, many before 1980, when the incidence of premature birth was less and fewer preterm newborns survived [1, 49], potentially artificially lowering effect estimates for the risk of cancer.

Our use of registry-based studies limits introduction of recall bias, but may be a source of selection bias. All studies retained in our project used population-based registries, which are well established in the United States and Scandinavia. Hence, most cases were Caucasian, and all were from developed nations. Moreover, the multifactorial nature of cancer development might translate into requiring a larger sample size to detect a change in disease risk. Thus, type II error may be present, despite our performing summary analysis when at least 200 combined cases were reported.

The limited sample sizes obtained for the occurrence of cancer in individuals born preterm induces another potential source of type II error. Indeed, the occurrence of malignancy among those born prior to 37 weeks gestation was rare, with hundreds to thousands of cases of the most common cancers in exposed individuals, despite millions of subjects being included in the combined studies for these cancers. Consequently, individual studies were under-powered to present the risk of cancer by degree of prematurity, as were we. For less common cancers, the order of magnitude of exposed cases was tens to hundreds, precluding quantitative analyses of these malignancies. Although the limited sample size is a limitation in determining the risk of cancer among those born preterm vs term, it may also be interpreted as a reassuring finding. If we assume that 10% of individuals are born preterm and that studies spanning decades yielded so few cases, then the absolute risk of cancer among persons born preterm is likely very small.

As previously mentioned, multiple studies have found increased risk of cancer among those born with low birth weight, including hepatoblastoma. Since birth weight varies as a function of GA, comparing low birth weight vs. normal birth weight infants does not discriminate between the impact of potential intra uterine growth restriction and GA on cancer development. Differentiating the effect of birthweight and GA on childhood cancer is challenging due to the limited data available. Ideally, we would compare the risk of hepatoblastoma among infants born weighing less than 10^th^ percentile for GA with those born at an appropriate weight for GA as a function of completed weeks of gestation at birth.

Our study suggests that prematurity has no impact on the incidence of most childhood cancers. Although the risk of hepatoblastoma may be increased, its occurrence is so rare that the National Cancer Institute’s surveillance program has been unable to calculate an incidence rate since 1999, when there were 5 to 10.5 cases per million children below five years of age[50, 51]. This decrease in cancer incidence is occurring despite increasing survival rates of preterm neonates. Furthermore, since parenteral nutrition has improved, and neonatal providers are more aggressive with enteral feed introduction, it is anticipated that the rate of hepatoblastoma in individuals born preterm may decrease.

## Conclusion

Preterm birth does not appear to be associated with an increased risk of most childhood cancers. Birth prior to 37 weeks GA may increase the risk of hepatoblastoma. Ongoing surveillance of aging prospective cohorts would be beneficial to provide insight into cancer risks in the aging population of individuals born preterm.

## Supporting information

S1 Table Search strategy. (DOCX)

S1 Fig Funnel plot for the ascertainment of publication bias in studies of all primary central nervous system tumors combined. (TIFF)

S2 Fig Funnel plot for the ascertainment of publication bias in studies of neuroblastoma. (TIFF)

S1 File MOOSE checklist. (DOC)

S2 File PRISMA checklist. (DOC)
