# Supplementary material for: Cancer risk in children and young adults born preterm: A systematic review and meta-analysis
Source: PLoS One. 2019 Jan 4;14(1):e0210366. doi: 10.1371/journal.pone.0210366 (PMC6319724; doi:10.1371/journal.pone.0210366)
Supplement: S1 Table — (DOCX) [file pone.0210366.s001.docx]

**S1Table. Search Strategy**

|  | **Search Term** | **Results** |
| --- | --- | --- |
| 2 | (cancer*):ti, ot, ab, tx, kw, ct, sh, hw, tn, dm, mf, dv, nm, kf, px, rx, an, ui | 4111109 |
| 3 | (malignan*):ti, ot, ab, tx, kw, ct, sh, hw, tn, dm, mf, dv, nm, kf, px, rx, an, ui | 983929 |
| 4 | (leukemia or leukaemia):ti, ot, ab, tx, kw, ct, sh, hw, tn, dm, mf, dv, nm, kf, px, rx, an, ui | 719134 |
| 5 | lymphoma.mp:ti, ot, ab, tx, kw, ct, sh, hw, tn, dm, mf, dv, nm, kf, px, rx, an, ui | 445390 |
| 6 | (tumor or tumour):ti, ot, ab, tx, kw, ct, sh, hw, tn, dm, mf, dv, nm, kf, px, rx, an, ui | 3675129 |
| 7 | *blastoma/ | 476 |
| 8 | *sarcoma/ | 40892 |
| 9 | *carcinoma/ | 84803 |
| 10 | #1 or #2 or #3 or #4 or #5 or #6 or #7 or #8 or #9 | 6704485 |
| 11 | (infant or child* or juvenile):ti, ot, ab, tx, kw, ct, sh, hw, tn, dm, mf, dv, nm, kf, px, rx, an, ui | 5152162 |
| 12 | ((prem* or preterm*) and birth):ti, ot, ab, tx, kw, ct, sh, hw, tn, dm, mf, dv, nm, kf, px, rx, an, ui | 125732 |
| 13 | #10 AND #11 AND #12 | 2273 |
| 14 | limit #13 to “all child (0 to 18 years)” [Limit not valid in CDSR, ACP, Journal Club, DARE, CCTR, CLCMR, Embase; records were retained] | 2092 |
| 15 | limit #14 to (english or french)[Limit not valid in CDSR, ACP, Journal Club, DARE, CLCMR, CLEED; records were retained] | 1990 |
| 16 | remove duplicates from #15 | 1672 |
