# Supplementary figures and images for: Cancer risk in children and young adults born preterm: A systematic review and meta-analysis
Source: PLoS One. 2019 Jan 4;14(1):e0210366. doi: 10.1371/journal.pone.0210366 (PMC6319724; doi:10.1371/journal.pone.0210366)

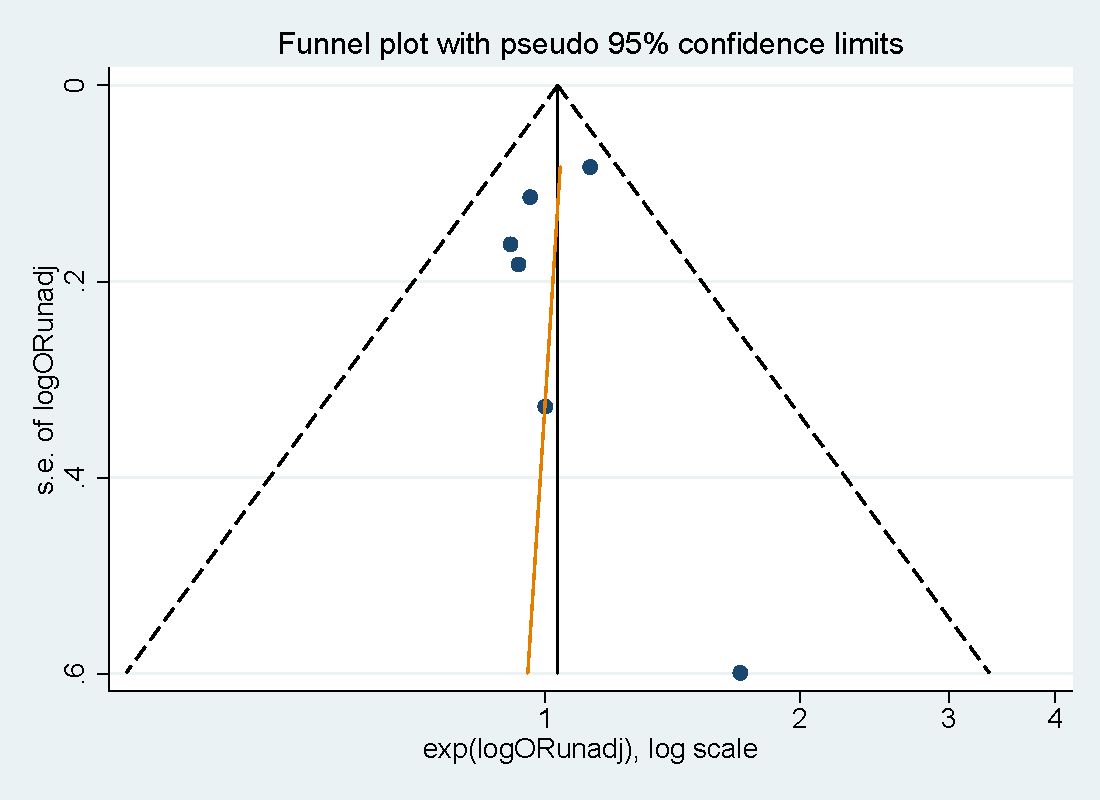

Supplement: S1 Fig — (TIFF) [file pone.0210366.s002.tiff]

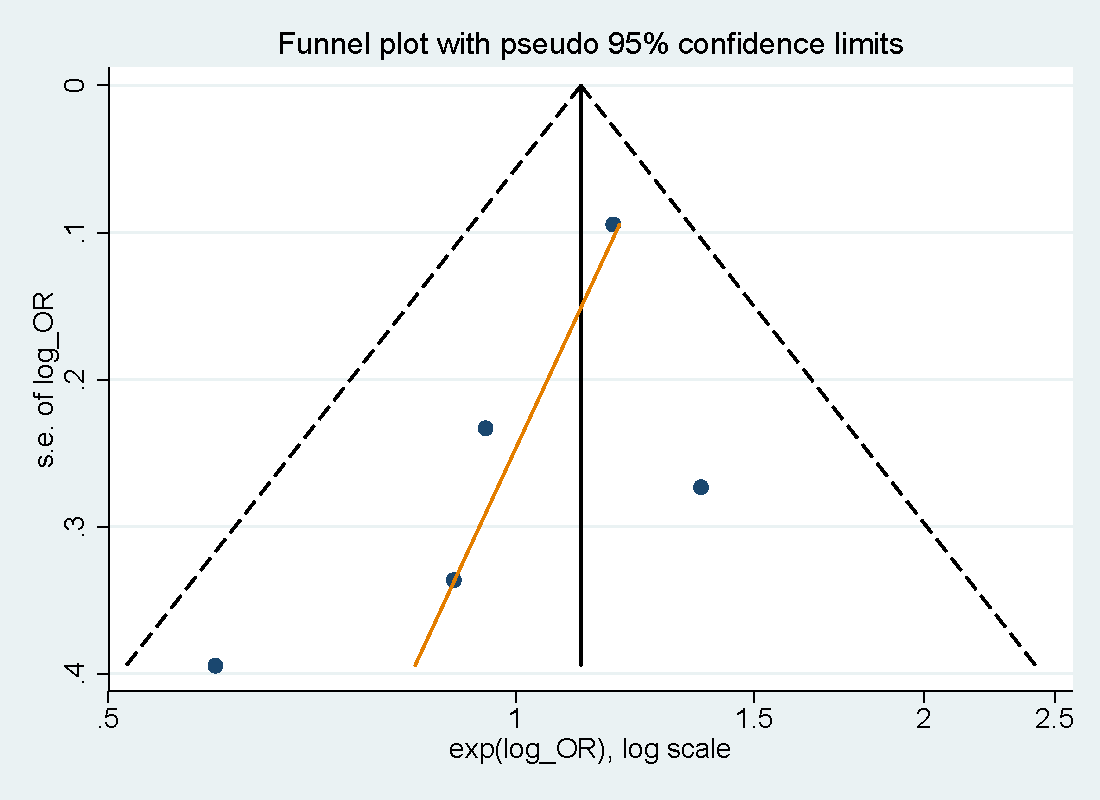

Supplement: S2 Fig — (TIFF) [file pone.0210366.s003.tiff]
